# Aromatase inhibitors in stimulated IVF cycles

**DOI:** 10.1186/1477-7827-9-85

**Published:** 2011-06-21

**Authors:** Evangelos G Papanikolaou, Nikolaos P Polyzos, Peter Humaidan, George Pados, Ernesto Bosch, Herman Tournaye, Basil Tarlatzis

**Affiliations:** 1 Human Reproduction and Genetics Foundation, Adrianoupoleos 6, 55133 Thessaloniki, Greece; 2 University Hospital, Dutch speaking Free University of Brussels, Laarbeeklaan 101, 1080, Brussels, Belgium; 3 The Fertility Clinic, Skive Regional Hospital, 7800 Skive, Denmark; 4 First Department of Obstetrics and Gynecology, Aristotle University of Thessaloniki, Perifereiaki Odos Thessalonikis-N. Efkarpias 564 29, Thessaloniki, Greece; 5 IVI, Valencia, Plaza de la Policía, Local 3, 46015 Valencia, Spain

## Abstract

Aromatase inhibitors have been introduced as a new treatment modality that could challenge clomiphene citrate as an ovulation induction regiment in patients with PCOS. Although several randomized trials have been conducted regarding their use as ovulation induction agents, only few trials are available regarding their efficacy in IVF stimulated cycles. Current available evidence support that letrozole may have a promising role in stimulated IVF cycles, either when administered during the follicular phase for ovarian stimulation. Especially for women with poor ovarian response, letrozole appears to have the potential to increase clinical pregnancy rates when combined with gonadotropins, whereas at the same time reduces the total gonadotropin dose required for ovarian stimulation. However, given that in all of the trials letrozole has been administered in GnRH antagonist cycles, it is intriguing to test in the future how it may perform when used in GnRH agonist cycles. Finally administration of letrozole during luteal phase in IVF cycles offers another treatment modality for patients at high risk for OHSS taking into account that it drastically reduces estradiol levels

## Background

Aromatase inhibitors are drugs traditionally used for the treatment of hormone responsive advanced breast cancer [1]. The last decade several reports have supported these agents and potential drugs for ovulation induction. Aromatase inhibitors inhibit the aromatization of androgens into oestrogens; in this regard, the hypothalamic-pituitary axis is released from the negative estrogenic feedback leading to increased follicular growth [2,3], whereas the increase of intraovarian androgens enhances early follicular growth may result in improved IVF outcome [4]. Furthermore, considering the short half life of these agents (~45 hours), their antiestrogenic effect during the late follicular phase is significantly reduced resulting improved endometrial thickness.

Several trials have tested the effect of AIs (letrozole or anastrozole) in women with anovulatory [5] or unexplained infertility [6], as a co-treatment in IVF/ICSI cycles, alone or in combination with other ovulation induction agents and in different treatment schedules or doses [7]. Despite the fact that these agents appear promising as ovulation induction agents, AIs have not been yet introduced in clinical practice, either because they do not appear to significantly enhance pregnancy rates compared to the current clinical practice, or simply due to the lack of large well designed randomized trials with positive results [8].

This lack of strong evidence is even greater regarding the use of AIs in IVF/ICSI cycles. Only few randomized trials, with limited series of patients, have been conducted up to date and the main research interest has been accumulated in the effect of letrozole in the treatment of poor responders.

## Follicular phase Aromatase Inhibitors use

### Normoresponders

Only one randomized trial has been conducted up-to-date that evaluated the addition of letrozole in patients with normal ovarian response undergoing IVF or ICSI [9]. Despite the fact that both implantation and ongoing pregnancy rates were higher in the letrozole co-treatment group the results were not significant different, owing mainly to the small sample size and the pilot nature of the study (Table 1). Nonetheless, letrozole co-treatment appeared to significantly augment endometrial thickness compared to FSH, an observation which may indeed explain both the increased implantation and ongoing pregnancy rates observed in these patients.

**Table 1:** Available randomized trials regarding the use of letrozole during the follicular phase in IVF/ICSI cycles

	Pituitary downregulation protocol/groups	Ovarian stimulation	Patients (N)	Clinical pregnancy rate (%)	Implantation rate (%)	Fertilization rate (%)	No oocytes (mean)	Total FSH dose (mean)
**Normoresponders**								
Verpoest 2006 [9]	Antagonist	rFSH+letrozole	10	50	31.25	63.3	13.8	1575
	Antagonist	rFSH	10	20	12.5	77.4	9.6	1650
**Poor responders**								
Goswami 2004 [10]	-	rFSH+letrozole	13	23	NA	NA	1.6	150
	Agonist	rFSH	25	24	NA	NA	2.1	2865
Garcia-Velasco 2005 [4]	Antagonist	rFSH+ HMG+ letrozole	71	22.4	25	68.2	6.1	3627
	Antagonist	rFSH+ HMG	76	15.2	9.4	63.3	4.3	3804
Ozmen 2009 [11]	Antagonist	rFSH+letrozole	35	28.6	NA	92.4	4.9	2980
	Antagonist	rFSH	35	17.1	NA	97.2	4.8	3850
Davar 2010 [12]	Antagonist	rFSH/HMG + letrozole	45	4.4	3.8	67.3	2.8	3158
	Agonist	rFSH or HMG	49	12.3	7.7	70.7	4.4	3458

N, number; NA, not data available

### Poor responders

Only four randomized trials have been published through 2010 with a total of 235 patients with poor ovarian response randomized to receive letrozole combined with gonadotropins or gonadotropins alone as ovarian stimulation protocols in IVF/ICSI cycles (Table 1). The gonadotrophin dose used was consistently lower in the letrozole co-treatment group in all of the trials.

The first small randomized trial published in 2004 examined the use of letrozole as part of a low-cost IVF protocol for poor responders. According to this study, letrozole+ rFSH resulted in comparable pregnancy rates with patients treated with a GnRH agonist and rFSH alone [10]. In addition in 2 trials in which pituitary downregulation in both treatment groups (gonadotropins alone or gonadotropins co-administed with letrozole) was performed with the use of a GnRH antagonist [4,11], letrozole co-treated patients experienced comparable pregnancy rates. On the contrary in a trial in which different GnRH analogues were used for downregulation, the administration of letrozole with FSH/HMG in a protocol using GnRH antagonists resulted in significantly lower implantation and fertilization rates, and significantly lower MII oocytes and top quality embryos compared to a microdose GnRH agonist protocol with FSH or HMG [12]. In accordance, a prospective, non-randomized, controlled trial [13] supported that ongoing pregnancy rates were significantly lower in the GnRH antagonist FSH+HMG+letrozole treatment group compared to GnRH agonist FSH+HMG group.

## Luteal phase aromatase inhibitors

The first randomized pilot study assessing the effect of administration of letrozole during the luteal phase of stimulated IVF cycles in oocyte donors was published in 2008 [14]. Despite the small number of patients included and the pilot design study serum estradiol levels were significantly lower in patients receiving letrozole compared to controls 4, 7 or 10 days after the hCG administration. Nonetheless, no difference in LH levels was observed among treatment and control groups (Table 2).

**Table 2:** Available randomized trials regarding the use of letrozole during the luteal phase in oocyte donors

	Luteal phase supplementation	Days of administration	Patients (N)	Serum estradiol levels (mean values)			LH levels (mean values)		
**LUTEAL PHASE**				Day 4	Day 7	Day 10	Day 4	Day 7	Day 10
Fatemi 2008 [14]	Letrozole 5 mg	For 14 days from OPU	3	272	229	31	0.2	0.1	0.1
	Placebo		3	749	1457	1308	0.2	0.1	0.1
Garcia-Velasco 2009 [15]	Letrozole 2.5 mg	For 5 days from OPU	15	279	240	40	0.21	0.18	0.40
	Placebo (folic acid)		15	1586	855	448	0.06	0.02	0.16

OPU, ovum pick-up

Another randomized placebo controlled trial has tested the same hypothesis in 30 egg donors after oocyte retrieval (Table 2). Administration of 2.5 mg letrozole for 5 days from the day of ovum pick-up, significantly reduced serum E2 levels at 4, 7 and 10 days after oocyte retrieval compared to placebo in accordance to the study by Fatemi et al. However, letrozole appeared to significantly increased LH levels on days 7 and 10 after retrieval [15].

Both randomized trials provided evidence that letrozole drastically reduces E2 levels whereas the trial Garcia-Velasco et al., demonstrated that letrozole restores LH secretion sooner in egg donors. In this regard AIs may be of interest not only for egg donors but also in patients at high risk of OHSS who freeze their oocytes/embryos or cancel hCG administration in order to reduce the risk linked to elevated estadiol levels.

## Aromatase inhibitors in cancer patients

The last five years letrozole has been introduced as an agent that could be used in cancer patients undergoing ovarian stimulation in order to preserve fertility prior chemotherapy, through embryo or oocyte cryopreservation [16]. The beneficial effect of letrozole has been underlined through several observation studies which supported that when used in ovarian stimulation it results in significantly lower estrogen exposure [17,18]. GnRh agonist triggering in these patients may also have beneficial effects when combined with stimulation with letrozole, since in results in significantly lower post-triggering estradiol levels [19]. However, the most important observation is that stimulation with letrozole and gonadotropins is unlikely to increase recurrence risk in cancer patients [20].

## Current practice and future implications

At the moment existing evidence for the use of aromatase inhibitors as part of an IVF/ICSI protocol is fragmented and weak. Despite the timeframe of ten years since introduction of AIs as ovulation induction agents, few randomized trials with limited number of patients have been conducted up to date. The lack of solid evidence in this field has been previously underlined [8], and definitely prevent us from introducing these evidence in clinical practice.

Based on the available data it appears that scant scientific evidence exists regarding the role of letrozole during the follicular phase women with normal ovarian response. However, letrozole may serve as an alternative for patients with poor ovarian response. A very important observation from the available data, is that a treatment protocol with GnRH antagonists, letrozole and gonadotrophins, hints a danger for compromising clinical pregnancy rates compared to treatment with gonadotropins in a microdose GnRH agonist protocol. Nonetheless, these negative results should not necessarily be attributed to letrozole, but may indeed be related to the type of analogue used. Microdose GnRH agonist flare protocols have been shown to be an effective treatment for poor responders, with randomized trials supporting significantly higher implantation rates compared to GnRH antagonist protocols [21,22]. Consequently, future trials may need to test whether AIs combined with gonadotropins may be comparable with gonadotropins alone in protocols involving GnRH agonists only. Taking into account that the dose of gonadotropins and the cost is significantly lower whenever letrozole is used, such an approach may be the most realistic cost effective way to treat women with poor ovarian reserve. Nonetheless, this needs to be replicated by large well designed trials.

Furthemore, the administration of letrozole during the luteal phase offers a new insight for patients at high risk for OHSS. Despite the fact that the results are based solely on one randomized trial, if they are confirmed by future studies letrozole may have a role as a preventive measure for OHSS.

Although a trial published by Tulandi et al. in 2006 [23] supported the safety of letrozole for the newborns, it should be noted that letrozole has not yet received an official permission in order to be administered in premenopausal patients as an ovulation induction agent. Therefore any use is off-label.

## Conclusions

Current evidence supports aromatase inhibitors can be a safe solution for fertility preservation in cancer patients prior chemotherapy. Although letrozole may have a role in the treatment of poor responder patients, results are based on few evidence and need to be replicated in the future, whereas its use in normoresponders requires even more intensive research. Their use should also include the long agonist protocol for IVF, as this protocol is widely used. Off label inscription shall be re-evaluated from pharmaceutical industry and reproductive associations in order more appropriate randomized studies to be performed.

## List of abbreviations

AIs: aromatase inhibitors; IVF: in vitro fertilization; ICSI: intracytoplasmic sperm injection; OHSS: ovarian hyperstimulation syndrome; HMG: human menopausal gonadotropin; FSH: follicular stimulating hormone; GnRH: gnonadotropin releasing hormone; LH: luteinising hormone; hCG: human chorionic gonadotropin.

## Competing interests

The authors declare that they have no competing interests.

## Authors' contributions

EGP wrote the manuscript, NPP wrote the manuscript, PH wrote the manuscript, GP, revised the manuscript, EB revised the manuscript, HT revised the manuscript, BT revised the manuscript. All authors read and approved the final manuscript.
